# miR-106b regulates the 5-fluorouracil resistance by targeting Zbtb7a in cholangiocarcinoma

**DOI:** 10.18632/oncotarget.17577

**Published:** 2017-05-02

**Authors:** Dechao Jiao, Yan Yan, Shaofeng Shui, Gang Wu, JianZhuang Ren, Yanli Wang, Xinwei Han

**Affiliations:** ^1^ Department of Interventional Radiology, The First Affiliated Hospital of Zhengzhou University, Zhengzhou, People's Republic of China; ^2^ Department of Oncology, The First Affiliated Hospital of Zhengzhou University, Zhengzhou, People's Republic of China; ^3^ Department of Interventional Radiology, The First Affiliated Hospital of Zhengzhou University, Zhengzhou, People's Republic of China

**Keywords:** MicroRNA-106b, chemoresistance, cholangiocarcinoma, 5-fluorouracil, Zbtb7a

## Abstract

**Background:**

Cholangiocarcinoma (CCA) is highly resistant to chemo-therapy, including 5-fluorouracil (5-FU) treatment. MicroRNAs are endogenous and short non-coding RNAs that can regulate multiple genes expression. Many microRNAs have shown functional roles in the chemo-resistance of tumors. Here, we examined the relationship between microRNAs expression and the sensitivity of CCA cells to 5-FU.

**Methods:**

Microarray analysis was used to determine the aberrantly expressed microRNAs in two 5-FU resistant CCA cell lines, KKU-M139 and KKU-M214 cells. To determine the effect of candidate microRNAs on 5-FU sensitivity, expression of candidate was modified via either transfection of a microRNA mimic or transfection of an antagonist. Ontology-based programs were also used to investigate the potential targets of microRNAs that were confirmed to affect the 5-FU sensitivity of CCA cells.

**Results:**

The microRNA-106b (miR-106b) was significantly down-regulated in 5-FU resistant CCA cells. Instead, over-expression of miR-106b could re-sensitize resistant CCA cells to 5-FU through down-regulation of Zbtb7a. Moreover, decreased expression of miR-106b is related to poor prognosis in patients with CCA, suggesting its potential role as a new prognostic marker in CCA.

**Conclusion:**

Our study demonstrates that miR-106b can reverse 5-FU resistance via Zbtb7a suppression, thus offer a novel and powerful strategy for CCA chemotherapy.

## INTRODUCTION

Cholangiocarcinoma (CCA) is one of the commonest malignant cancers around the word, and CCA-related death abruptly increased in the past decades [1]. Because of lacking the effective screening biomarkers, CCAs are often diagnosed along with lymph-node metastases [2]. Depressingly, almost all CCAs are resistant to chemotherapies, drug-resistance makes almost all CCA patients tending to be recurrence after surgical resection [3]. And the chemo-resistant rapidly increased the morbidity and mortality rates for CCA worldwide. Therefore, to clearly elucidate the mechanism of chemo-resistant in CCA and to enhance CCA tumors sensitive to chemotherapies remain an urgent requirement.

5-fluorouracil (5-FU) is a commonly used drugs for many types of tumors. Though multi-types of tumors are benefit from 5-FU treatment, the overall response rate in CCA patients to 5-FU is limited [4]. 5-FU resistance in many types of cancers is due to aberrantly expression of many oncogenes involving many processes in tumorigenesis, including cell proliferation, metastasis or metabolism [5]. 5-FU is the most commonest drugs for CCA treatment, to clarify the molecular mechanism of 5-FU resistance remains urgent requirement.

MicroRNAs (miRNAs) are small non-coding RNAs that modulate the target gene expression by directly binding to 3′UTR and therefore suppress the mRNA transcription. Functional studies have shown miRNAs to participate in almost every cellular process including apoptosis, proliferation and differentiation by directly modulating the expression of tumor suppressor genes and oncogenes [6]. Multiple microRNAs have been reported to regulate the progression, tumorigenesis and chemoresistance of CCA [7–11]. Therefore, it is urgent to identify and clarify the functional microRNAs in CCA.

In this study, we identified a novel aberrantly expressing microRNA, miR-106b, to modulate the 5-FU sensitivity of CCA cells through suppressing Zbtb7a expression.

## RESULTS

### MiR-106b is downregulated in 5-FU resistant CCA cells and human tumor tissues

**Figure 1:**
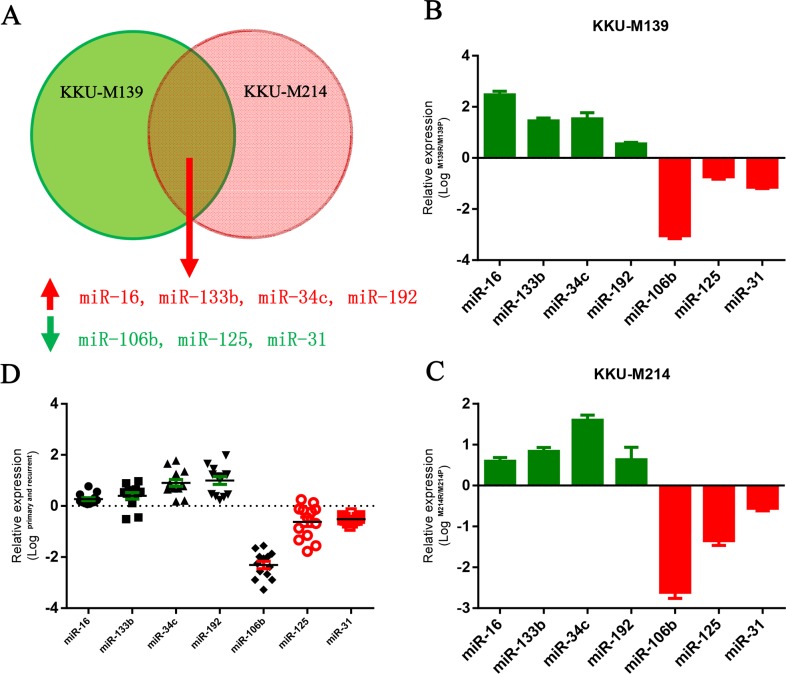
MiR-106b is downregulated in 5-FU resistant CCA cells and human tumor tissues **(A)** Dysregulated microRNAs in 5-FU resistant KKU-M139 and KKU-M214 cells identified by microarray. **(B and C)** Aberrantly expressed microRNAs in KKU-M139 **(B)** and KKU-M214 **(C)** cells were confirmed by q-PCR. **(D)** The aberrantly expressed microRNAs were confirmed by q-PCR in primary or recurrent CCA tumors. qRT-PCR results were normalized by GAPDH. All data was shown as mean ± SEM from three independent experiments.

To identify aberrantly expressed microRNAs in the development of chemo-resistance, we used two 5-FU resistant CCA cells to perform a microarray analysis to compare miRNA expression changes in 5-FU resistant and sensitive CCA cells (Supplementary Figure 1). Four down-regulated microRNAs and four up-regulated microRNAs were identified as differentially expressed (>=5-fold) in both KKU-M139 cells and KKU-M214 cells (Figure 1A). These aberrantly expressed microRNAs were validated by quantitative PCR in both M139 and M214 cells (Figure 1B and 1C). Furthermore, we also sought to determine whether these microRNAs were aberrantly expressed in clinical CCA samples. The expression of these microRNAs in tumor samples obtained from matched pairs of primary and recurrent 5-FU-refractory CCA from 18 patients. Among them, we notified that miR-106b was the most aberrantly down-regulated microRNAs in clinical samples (Figure 1D). Therefore, we chosen miR-106b for subsequent studies.

### miR-106b re-sensitizes CCA cells to 5-FU

**Figure 2:**
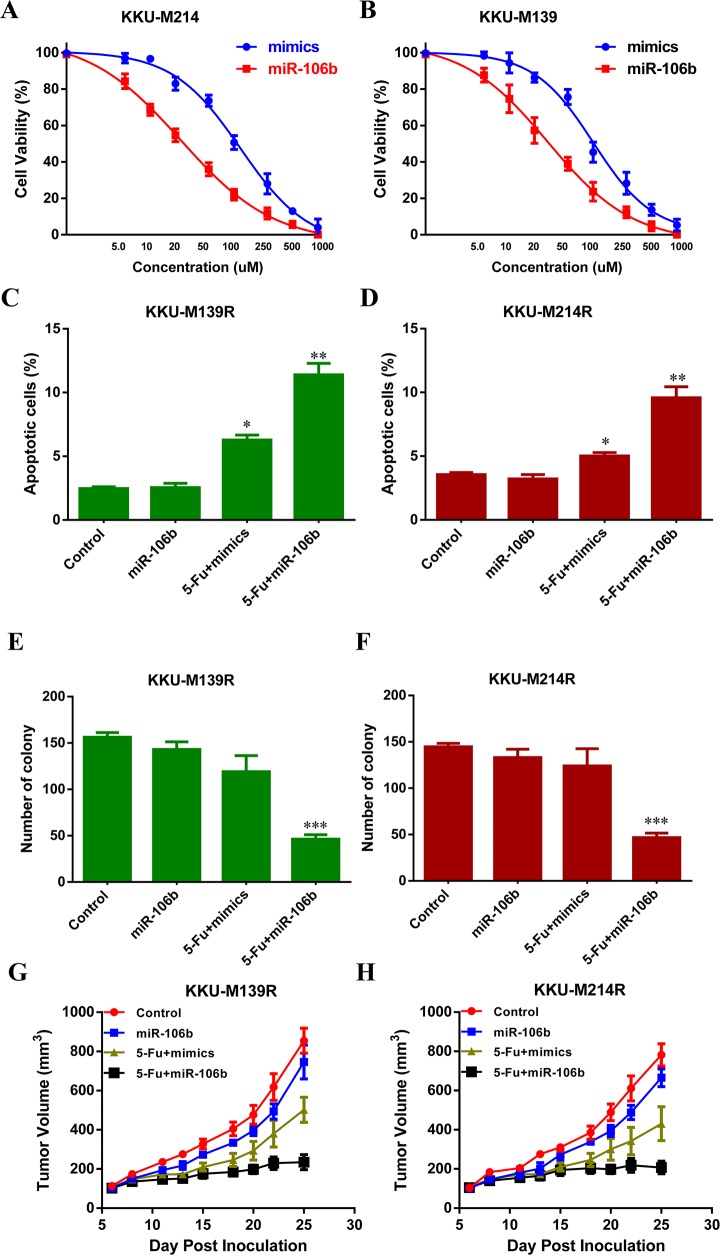
miR-106b re-sensitizes CCA cells to 5-FU **(A and B)** 5-FU-resistant M139 **(A)** and M214 **(B)** cells were transduced with miR-106b mimics and then treated with different dose of 5-FU and the percentage of cell survival was examined by MTT assays. **C and D**. The apoptotic rate of M139 **(C)** and M214 **(D)** cells, which were transduced with miR-106b mimics and then treated with 40 μM 5-FU for 36 h, were examined by flow cytometry. **(E and F)** Colony formation assay showed the numbers of colonies of M139 **(E)** and M214 **(F)** cells transduced with miR-106b and then treated with 40 μM 5-FU. **(G and H)** Tumor volume of subcutaneous xenografts, which were inoculated with M139 **(G)** or M214 **(H)** CCA cells, were measured. All data represent the means ± SEM of three independent experiments. * represents p<0.05, ** represents p<0.01, *** represents p<0.001.

To explore whether miR-106b could re-sensitize the CCA cells to 5-FU, KKU-M139R (M139R) cells and KKU-M214R (M214R) cells were transduced with miR-106b mimics and then treated with different dose of 5-FU. Cell viability analysis showed that forced miR-106b expression rendered these two cell lines more sensitive to 5-FU (Figure 2A and 2B). The half maximal inhibitory concentration (IC50) values of M139R and M214R cells are about 42.5 uM and 38 uM, respectively (Figure 2A and 2B). As expected, miR-106b over-expression re-sensitized CCA cells to 5-FU (Figure 2C and 2D). Flow cytometry analysis also showed that miR-106b overexpression significantly induced more apoptotic M139R and M214R cells (Figure 2E and 2F). We further performed colony formation assay. As shown in Figure 2G and 2H, in the absence of 5-FU, miR-106b alone could decrease the number of colonies of M139R and M214R cells (Figure 2G and 2H). These results showed that suppression of miR-106b might render CCA cells resistant to 5-FU *in vitro*. To evaluate the effect of miR-106b on 5-FU resistance *in vivo*, stably miR-106b overexpressing M139R and M214R cells were injected subcutanenously into two groups of nude mice. The mice were also injected intraperitoneally with 30 mpk 5-FU every 5 days after the tumor volume reaching 100 mm^3^. As expected, the tumor growth was significantly reduced by miR-106b overexpression (Figure 2I and 2J). These results indicated that miR-106b sensitized resistant CCA cells to 5-FU *in vivo*.

### Zbtb7a is the direct target of miR-106b in CCA cells

**Figure 3:**
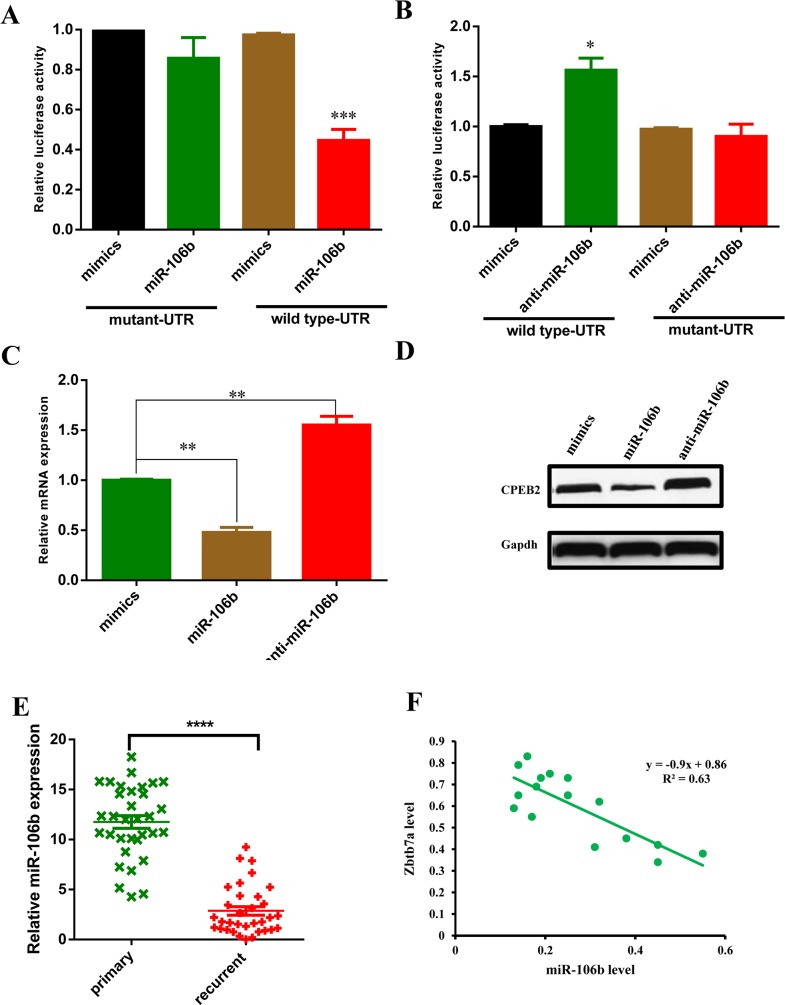
Zbtb7a is the direct target of miR-106b **(A and B)** Luciferase activity of the reporter construct containing wild type or mutant 3′UTR of Zbtb7a was measured after co-transfection with 100 nM miR-106b mimics **(A)** or anti-miR-106b mimics **(B)**. **(C)** The Zbtb7a mRNA level was measured in M139 cells after transfection with 100 nM miR-106b mimics or anti-miR-106b mimics. **(D)** The Zbtb7a protein level was measured by western blot assay in M139 cells after transfected with miR-106b mimics and anti-miR-106b mimics. **(E)** The mRNA expression level of Zbtb7a in primary CCA tumors and recurrent CCA tumors was examined by q-PCR. **(F)** The correlation of miR-106b and Zbtb7a in CCA recurrent tumors was measured by q-PCR. All data represent the means ± SEM of three independent experiments. * represents p<0.05, ** represents p<0.01, *** represents p<0.001.

To investigate how miR-106b modulate CCAs sensitive to 5-FU, we performed bioinformatics analysis by using the publicly acailable database miRWalk. Zbtb7a was predicted to be one of the potential target genes of miR-106b. Next, we constructed luciferase reporter plasmids which contained wild type or mutant Zbtb7a 3′-UTR. Luciferase reporter assays demonstrated that exogenous miR-106b repressed the luciferase activity of wild type 3′-UTR of Zbtb7a, but not the luciferase activity of mutant 3′-UTR of Zbtb7a (Figure 3A). In contrast, inhibition of miR-106b with miR-106b antagonist increased the activity of luciferase reporter fused to the wild type 3′-UTR but not the mutant UTR (Figure 3B). Furthermore, forced miR-106b mRNA (Figure 3C) and protein (Figure 3D) level in M214R cells reduced Zbtb7a expression, while miR-106b suppression increased Zbtb7a expression. To confirm the above findings, we analyzed the correlation between miR-106b and Zbtb7a in clinical CCA samples. The expression of miR-106b was found to be inversely correlated with Zbtb7a in clinical CCA samples (Figure 3E). The patient characteristics were summarized as described in Table 1AQ 1. Taken together, these results suggested that Zbtb7a was a direct target of miR-106b in CCA cells.

**Table 1:** Clinical characteristics of CCA patients

Characteristics	Patient number(n=66)	miR-106b high (n=33)	miR-106b low (n=33)	*P*-value
**Age, years**				0.653
>=60	41	20	21	
<60	25	13	12	
**Gender**				0.472
Male	44	21	23	
Female	22	12	10	
**CA19-9 level (U/L)**				0.237
<=37	38	18	20	
>37	28	15	13	
**Tumor diameter (cm)**				0.316
< 3	27	16	11	
>= 3	39	17	22	
**Lymph node metastasis**				0.061
Yes	32	23	9	
No	34	10	24	
**Differentiation**				0.581
Well/moderate	37	25	12	
Poor	29	8	21	
**Neural invasion**				0.084
Yes	44	18	26	
No	22	15	7	

The clinical characteristics were analyzed by using chi-square test.

### Zbtb7a is the functional target of miR-106b in modulating CCA cells sensitive to 5-FU

**Figure 4:**
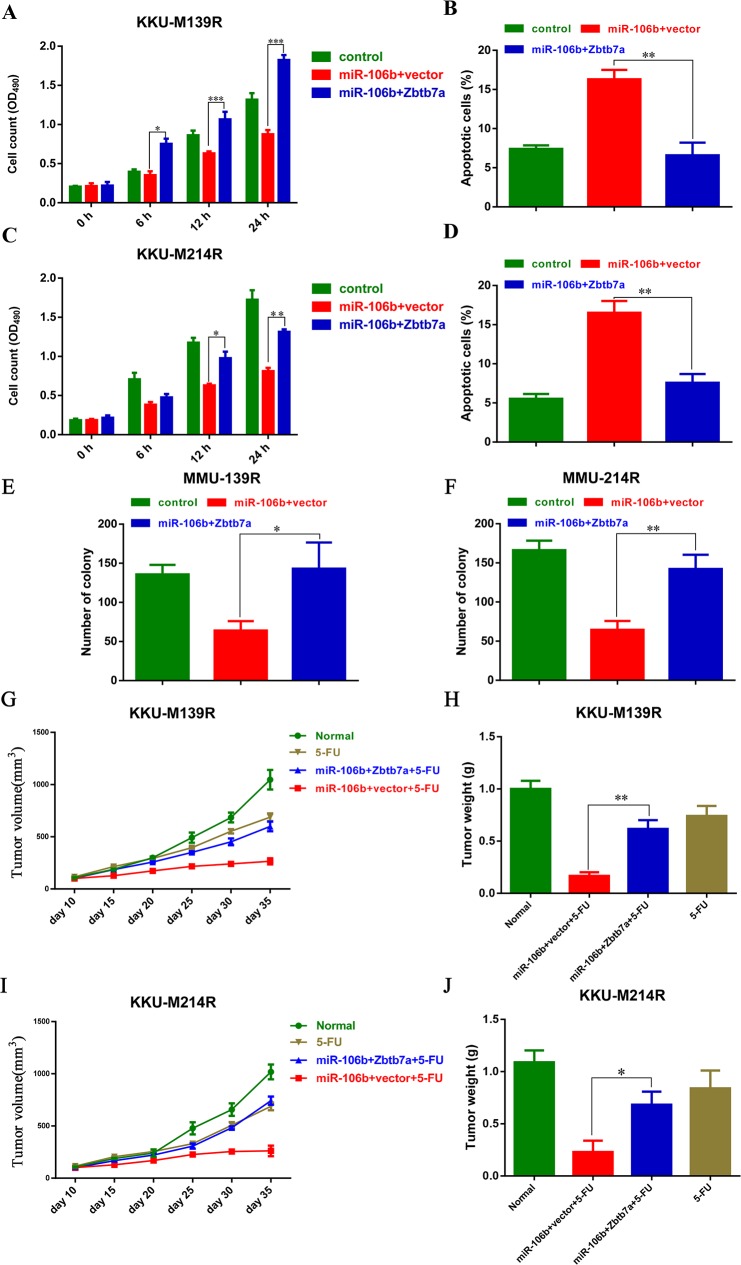
Zbtb7a is the functional target of miR-106b in modulating CCA cells sensitive to 5-FU **(A and C)** The growth of M139 **(A)** and M214 **(C)** cells, which were stably expressing miR-106b and Zbtb7a, were measured by MTT assays. **(B and D)** The apoptotic M139 **(B)** and M214 **(D)** cells were examined by flow cytometry after these cells were transduced with miR-106b and Zbtb7a and then treated with 40 μM 5-FU. **(E and F)** Zbtb7a rescues colony formation of MMU-139 and MMU-214 cells. **(G and I)** Stably miR-106b and Zbtb7a expressing M139 **(G)** and M214 cells **(I)** were inoculated into BALB/c nude mice. Tumor volume was measured every 5 days when the tumor reaching 100 mm^3^. **(H and J)** Tumor weight was measured at the end point of these experiments. All data represent the means ± SEM of three independent experiments. * represents p<0.05, ** represents p<0.01, *** represents p<0.001.

To further evaluate the functional role of Zbtb7a in miR-106b-mediated chemo-resistance, we over-expressed Zbtb7a in these two CCA cells transfected with miR-106b, and treated those cells with 5-FU. The MTT assays showed that Zbtb7a overexpression re-sensitized M139R cells and M214R cells to 5-FU (Figure 4A and 4B). Consistently, the apoptotic cells were also reduced by Zbtb7a overexpression in these two CCA cell lines (Figure 4C and 4D). Extensively, the colony formation assays were also performed. As expected, the colony formation of CCA cells were restored by Zbtb7a overexpression (Figure 4E and 4F). Moreover, Zbtb7a overexpression also restored the tumor growth *in vivo* (Figure 4G-4J). These results suggested that Zbtb7a is the functional target of miR-106b in modulating CCA sensitive to 5-FU.

## DISCUSSION

Cholangiocarcinoma (CCA) is one of the most common primary hepatobiliary cancer [12]. Though a majority of patients are benefit from surgical resection, the curative therapy for CCA is still unavailable [2]. In addition, most CCA patients are unable to surgical resection at the time of diagnosis [13]. On the other hand, a majority of CCA patients feel desperate due to chemo-resistance and recurrence [14]. Multidrug resistance (MDR) is a major reason to recurrence. Therefore, to clarify the mechanism how CCA cells defense the 5-FU cytotoxicity is urgently needed. In this study, we identified several aberrantly expressed microRNAs in 5-FU resistant CCA. Our results revealed that miR-106b was significantly downregulated in 5-FU resistant CCA cell lines and 5-FU resistant clinical tumor samples. Thus, decreased expression of microRNA-106b might be used as a prognostic molecular marker.

MiR-106b is known to promote cancer cell proliferation and survival in gastric cancer and hepatocellular carcinoma [15–17]. MiR-106b has been reported to regulate chemo-resistance by suppressing the expression of EP300 [18]. It also regulates colorectal cancer cell resistant to radiotherapies by PTEN/PI3K/AKT signaling [18]. The miR-106b∼25 cluster also promotes bypass of doxorubicin-induced senescence and increase in motility and invasion by targeting the E-cadherin transcriptional activator EP300 [19]. *Koster R* has reported that Oct4/miR-106b/p21 axis offered new strategies for the treatment of chemoresistant testicular and other types of cancer [20]. In this paper, our results showed that miR-106b significantly reduced in 5-FU resistant cells and 5-FU resistant tumors and modulated the progression and tumorigenesis of CCA via suppressing Zbtb7a expression. However, whether other members of miR-106b-25 cluster also contributes the development of drug resistance are not clear.

Recently, Zbtb7a was characterized as an oncogene in many types of cancers, which represses tumor suppressor ARF gene transcription. Zbtb7a expression in several type of cancers is remarkably higher than its in normal tissues [21–24]. In this study, we find that Zbtb7a is the direct target of miR-106b in CCA cells. Furthermore, forced Zbtb7a expression restored miR-106b-induced cell sensitive to 5-FU. Moreover, Zbtb7a overexpression also reduced the 5-FU sensitivity in CCA xenograft tumors. These results suggested that Zbtb7a was the direct and functional target of miR-106b in CCA cells.

In summary, this study identified a novel microRNA, miR-106b, which acted as a suppressor of chemoresistance in CCAs. Our results also suggested that Zbtb7a was the direct and functional target of miR-106b to modulate the 5-FU resistance in CCA cells. The miR-106b/Zbtb7a axis could affect chemotherapy sensitivity of CCA and miR-106b also provided a new target for CCA treatment.

## MATERIALS AND METHODS

### Clinical samples

The primary and recurrent samples were obtained at the First Affiliated Hospital of Zhengzhou University from 2011 to 2016. Tissues were flash frozen immediately after surgery. These samples were collected at the time of diagnosis. The study was approved by the Research Ethics Committee of Zhengzhou University, and written informed consent was obtained from all participants.

### Microarray analysis

Total RNAs were extracted using TRIzol reagent (Invitrogen) according to the manufacturer's description, quantified using the NanoDrop ND-1000 and the RNA integrity was assessed using standard denaturing agarose gel electrophoresis, purified using the miRVana miRNA Isolation Kit (Ambion, Austin, TX, USA), tailed with polyadenylation polymerase, ligated with biotinylated DNA dendrimers, and hybridised to Affymetrix GeneChip miRNA arrays using the FlashTag Biotin RNA Labeling Kit (Genisphere, Hatfield, PA, USA) according to the manufacturer's instructions. Slides were scanned with the Affymetrix GeneChip Scanner 3000 (Affymetrix, Santa Clara, CA, USA), and miRNA data were analysed using the miRNA QC Tool (Affymetrix).

### Cell culture

KKU-M214P/R cells and KKU-M139P/R cells were cultured in RPMI 1640 culture medium supplemented with 10% fetal bovine serum, 2 mM L-glutamine, 100 mg/mL streptomycin and 100 U/mL penicillin in an incubator with a humidified atmosphere and 5% CO_2_ at 37°C. Transfection was performed using Lipofectamine 2000 reagent (Invitrogen, Carlsbad, CA, USA) according to the manufacturer's instructions as previously described. The miR-16b mimics, miR-16b antagonist and their scramble controls were synthesized by Sangon (Shanghai, China).

### Real-time PCR

Total RNAs were extracted from cancer cells by using RNAiso Plus reagent (Takara Biotechnology Co., Ltd, DALIAN). To detect miR-106b expression, total RNAs were reversed using MMLV reverse transcriptase. The resultant cDNA was then used as template to perform real time PCR using a real time PCR kit (Qiagen). Transcripts were quantified by real time PCR and normalized to the amount of U6 mRNA expression. For Zbtb7a mRNA expression analysis, first strand cDNA was synthesized by using cDNA synthesis kit (Takara) according to the manufacturer's instructions. Their expression at mRNA level were detected by using Syber Green PCR mastermix (Applied Biosystems). All primers were listed at Table 2.

**Table 2:** The sequences of primers used in this study

Name		Sequence
miR-106b	RT	5′CTCAACTGGTGTCGTGGAGTCGGCAATTCAATCTGCACTGTCA 3′
	Forward	5′ACACTCCAGCTGGGTGGCATAAAGTGCT 3′
	Reverse	5′CTCAACTGGTGTCGTGGA 3′
Actin	Forward	5′AGCCTCAAGATCATCAGCAATGCC 3′
	Reverse	5′TGTGGTCATGAGTCCTTCCACGAT 3′
Zbtb7a	Forward	5′ATCTGCGAGAAGGTCATCCA 3′
	Reverse	5′CAGCAGCTGTCGCACTGGTA 3′
U6	Forward	5′’CTCGCTTCGGCAGCACA 3′
	Reverse	5′AACGCTTCACGAATTTGCGT 3′

### Luciferase reporter assays

The DNA oligonucleotide and the pMiR-Reporter Vector were used to build the luciferase report vectors (pMiR-Zbtb7a-wild type and pMiR-Zbtb7a-Mutant). HEK293T cells were co-transfected with pMiR-Zbtb7a-wild type or pMiR-Zbtb7a-Mutant and miR-106b or miR-106b antagonist mimics. A Renilla luciferase-expressing plasmid pRL-TK (Promega) used as control was also co-transfected. Cells were harvested and luciferase activity was determined using the Dual Luciferase Reporter Assay Kit (Promega) at 24 h after transfection. The results are expressed as relative luciferase activity (firefly luciferase/Renilla luciferase).

### MTT assays

CCA cells were seeded in 96-well plates (4×10^3^ cells per well) and at 37°C in an incubator containing 5% CO_2_. Cells were treated with 40 μM 5-FU, 40 μM 5-FU plus miR-106b mimics (Genepharma, Suzhou, China). Cell viability was tested by using 3-(4,5-Dimethyl-2-thiazolyl)-2,5-diphenyl-2H-tetrazolium bromide (MTT, Sigma) assay at 0 h, 12 h, 24 h after treatment. Briefly, cells were incubated with MTT at a final concentration of 0.5 mg/mL for 4 h. The supernatant was discarded, and the precipitated formazan was dissolved in dimethyl sulfoxide. Absorbance was measured at 490 nm with micro-plate reader (Molecular Devices, i3).

### Western blotting

CCA cell lysates were prepared with RIPA Lysis buffer (Beyotime, China) containing protease inhibitor cocktail (Roche). Protein samples were loaded for sodium dodecylsulfate-polyacrylamide gel electrophoresis (SDS-PAGE) and transferred to a nitrocellulose membrane. After a blockage of 5% fat-free milk, the membrane was probed with primary anti-Zbtb7a (dilution 1:1000, Santa Cruz Biotechnology) and anti-GAPDH (dilution 1:2,000, Santa Cruz Biotechnology). After washing, the membrane was incubated with horseradish peroxidase-conjugated (HRP) secondary antibody (1:2000, Santa Cruz Biotechnology) for 1 h. The signal was visualized using the ECL detection system (Thermo Fisher, USA) and quantified by densitometry using Quantity One software (Bio-Rad, Hercules, CA, USA).

### Flow cytometry

CCA cells treated with 5-FU, 5-FU plus miR-106b mimics for flow cytometry analysis using an Annexin V Apoptosis Detection Kit (Becton Dickinson, NJ, USA), untreated group was considered as control. Cells were stained with Annexin V-fluorescein isothiocyanate (FITC), propidium iodide (PI) for 25 min, and then analyzed by flow cytometry (BD CantoII). FACS data were analyzed using FlowJo (Tree Star, Inc.).

### Colony formation assay

1.0*10^3^ cells, which were transduced with miR-106b or control mimics were seeded into 6-well plates (in triplicates) in 2 ml of complete growth medium. The medium of each well was changed every three days. Two to three weeks later, cells were stained by 0.1% crystal violet (Sigma-Aldrich, St. Louis, MO, USA) in methanol for 10 min. The colonies more than 50μm were counted directly on the plate. Statistical significance was calculated from at least three independent experiments.

### Tumor xenograft models

Six to eight-week-old BALB/c (nu/nu) mice were purchased from Shanghai SLAC Laboratory Animal Co. All mice were maintained in a barrier facility at Animal Center of Chongqing Medical University. 2.0*10^6^ stably expressing miR-106b or scramble control cells were implanted subcutaneously (s.c.) into the right flank of mice. The tumor bearing mice were subject to 30 mpk 5-FU (i.p.). Tumor volume was measured every 5 days when the tumor volume reaching 100 mm^3^. All groups of mice were sacrificed and tumors were weighted at the endpoint of this experiments.

### Statistical analysis

Data were presented as mean ± SEM. Group comparison was performed by Student's t-test. P value <0.05 was considered as significant difference. *, **, and *** donates significance at 0.05, 0.01 and 0.001 level respectively.

## SUPPLEMENTARY MATERIALS FIGURES AND TABLES


