# Supplementary material for: miR-106b regulates the 5-fluorouracil resistance by targeting Zbtb7a in cholangiocarcinoma
Source: Oncotarget. 2017 May 2;8(32):52913–22. doi: 10.18632/oncotarget.17577 (PMC5581081; doi:10.18632/oncotarget.17577)
Supplement: Supplementary file 1 [file oncotarget-08-52913-s001.pdf]

miR-106b regulates the 5-fluorouracil resistance by targeting Zbtb7a in cholangiocarcinoma

SUPPLEMENTARY FIGURE

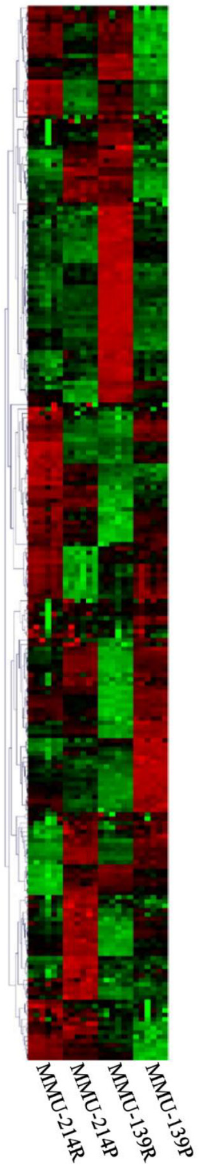

Supplementary Figure 1: The microarray analysis to identify aberrantly expressed microRNAs in of 5-FU resistant CCA cells.
